# Intra- and Cross-Species Transmission of Astroviruses

**DOI:** 10.3390/v13061127

**Published:** 2021-06-11

**Authors:** Shanley N. Roach, Ryan A. Langlois

**Affiliations:** 1 Biochemistry, Molecular Biology, and Biophysics Graduate Program, University of Minnesota, Minneapolis, MN 55455, USA; roach138@umn.edu; 2 Department of Microbiology and Immunology, University of Minnesota, Minneapolis, MN 55455, USA

**Keywords:** astrovirus, virus transmission, recombination, cross-species transmission, zoonotic potential

## Abstract

Astroviruses are non-enveloped, single-stranded RNA viruses that infect mammalian and avian species. In humans, astrovirus infections are one of the most common causes of gastroenteritis in children. Infection has also been linked to serious neurological complications, especially in immunocompromised individuals. More extensive disease has also been characterized in non-human mammalian and avian species. To date, astroviruses have been detected in over 80 different avian and mammalian hosts. As the number of hosts continues to rise, the need to understand how astroviruses transmit within a given species as well as to new host species becomes increasingly important. Here, we review the current understanding of astrovirus transmission, the factors that influence viral spread, and the potential for cross-species transmission. Additionally, we highlight the current gaps in knowledge and areas of future research that will be key to understanding astrovirus transmission and zoonotic potential.

## 1. Introduction

Astroviruses (AstVs) are small, non-enveloped viruses with a single-stranded, positive sense RNA genome. AstVs are among the most common causes of gastroenteritis in children after norovirus and rotavirus infections [1]. Although primarily associated with asymptomatic or diarrheal disease in humans, central nervous system complications and more serious disease, especially in immunocompromised individuals, have been reported [2,3]. Broader disease has also been documented in non-human mammalian and avian species, including enteritis, hepatitis, nephritis, and neurological symptoms [4,5,6,7,8]. The presence of AstV in farm animals has both economic and public health implications. Asymptomatic infection allows transmission within a population, while more severe infections can result in stunted growth, decreased harvests, and death. Human interaction with infected farm animals and consumption of AstV contaminated animal products provide avenues for zoonotic transmission and are thus public health concerns.

**Figure 1:**
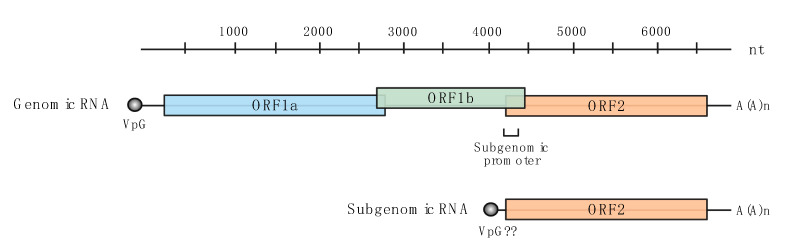
Schematic representation of astrovirus genomic and subgenomic RNA organization. Genomic RNA is connected to the viral genome-linked protein (VpG) at the 5′ end (grey); subgenomic RNA may have a VpG but this has yet to be confirmed [13,14,15]. Three reading frames encode the viral proteins: proteases and VpG in ORF1a (blue), the RdRP in ORF1b (green), the capsid, spike and structural proteins in ORF2 (orange). A poly-A tail is encoded in the genome at the 3′ end.

AstVs are broken into two genera, mamastroviruses (MAstVs) and avastroviruses (AAstVs), based on whether they infect mammalian or avian species, respectively. Based on nucleic acid and amino acid sequences, MAstVs and AAstVs diverged approximately 310 million years ago [9], but the structure of the genome is conserved between MAstVs and AAstVs. Ranging from 6.8 to 7.9 kilobases in length, the genome can be divided into the following components: A 5′ untranslated region (UTR), the open reading frames (ORFs), and a 3′ UTR and poly-A tail that is genomically encoded (Figure 1) [10,11]. ORF1a and ORF1b encode for the viral non-structural proteins, including the viral genome-linked protein (VpG) and the RNA-dependent RNA polymerase (RdRP), respectively. ORF2 encodes for the viral capsid, spike, and structural proteins and is also expressed as subgenomic RNA during replication [11]. ORF1b is the least divergent region between species and serotypes of viruses, whereas ORF2 is the most divergent and is used to determine phylogeny between viruses [12].

AstVs can infect a multitude of hosts and have been detected in over 80 different avian and mammalian host species (Table 1) [8]. Among those species susceptible to AstV infection are humans, domestic animals such as pigs and turkeys, and wild animals including multiple rodent and bat species [4]. There are three divergent groups of human AstVs (HAstVs): classic, HAstV-MLB, and HAstV-VA/HMO groups (Table 1) [16]. The classical group of HAstVs, comprised of eight serotypes (HAstV1-8), can be grown in cell culture and are the most well studied. Recent discoveries have expanded the host range for AstVs, demonstrating infections in marine mammals such as bottlenose dolphins, terrestrial animals like the cheetah, and a diverse range of avian species such as the European roller and black-naped monarchs [17,18,19,20]. AstVs have also been recently found in fish. These AstV sequences do not form their own genera of AstVs, but rather are distributed throughout both MAstV and AAstV phylogenies and are currently unclassified (Table 1) [21]. This suggests a history where various AstVs jumped between avian or mammalian species and fish. Additionally, the first AstV infections in invertebrates was recently found. These astro-like viruses identified in insects were phylogenetically distinct from vertebrate AstVs [22]. The large number of recently identified and largely unclassified viruses, both in new vertebrate species as well as invertebrates, indicate there is an extremely broad AstV host range that has yet to be fully characterized and appreciated. This also suggests that there are many more AstVs yet to be discovered. This increasing diversity has created challenges in determining the classification scheme and evolutionary distance between AstVs. Since their discovery, the classification scheme has been modified numerous times [23,24]. The ninth report from The International Committee on the Taxonomy of Viruses (ICTV) in 2011 signaled the field is moving away from classifying MAstVs and AAstVs based solely on host species [24]. The continued discovery of new AstVs will necessitate continued updating of classification and evaluation of the evolutionary distance and relationships between AstVs.

Despite the advances made recently and the improvement of sequencing technologies in characterizing and discovering AstVs, many hurdles and unknowns remain in AstV research. For example, the mechanisms of cell entry and cell tropism have not been well characterized. While some light has been shed recently on the tropism of enteric human and murine AstVs [68,69,70], the tropism for AstVs in general—especially AAstVs—remains to be determined. In this review, we summarize the current understanding of the mechanisms of transmission, mutation and recombination, cross-species transmission, and highlight areas of future research in AstV transmission.

## 2. Mechanisms of Transmission

AstVs are primarily transmitted fecal-orally through either direct interaction with feces or the consumption of contaminated food or water. AstVs transmit efficiently in both terrestrial and aquatic environments. Both avian and mammalian species that congregate in large groups or live in highly dense populations provide highly permissive environments for AstV transmission. Avian migrations provide the opportunity for AstVs from different geographical regions to be introduced into new areas and populations of hosts. In terrestrial animals, interaction with and the inspection of feces is very common. AstVs readily transmit between coprophagic animals such as rodents. Transmission between commercially farmed animals is extremely common and compounded by persistent viral shedding from infected animals long after the date of initial infection [8,66]. This persistent viral shedding also increases the risk of spill over into farm animals of different species. In wild animals, feces serve many roles, including marking territories, attracting mates, hunting prey, and avoiding predators. These various interactions facilitate intra-species transmission as well as open the door for cross-species exposure.

Unlike enveloped viruses such as coronavirus or influenza, which are sensitive to lipid solvents and changes in pH or temperature due to their lipid membranes, non-enveloped viruses like AstVs are very stable in the environment and persist long-term in water, feces, and other materials [8,71,72,73]. One study found HAstV8 was able to remain infectious in surface and ground water for two and six months, respectively [74]. Another group observed that incubation of HAstVs with bacteria and bacterial components such as lipopolysaccharides preserved virus infectivity by stabilizing the viral capsid [75]. They also observed that mucin, the major component of mucus, also stabilized the capsid of HAstVs [75]. Stabilization of the capsid allows AstVs to remain infectious for longer periods of time after shedding, increasing the likelihood that a shed virus will be able to encounter and infect new hosts. This has far reaching implications for spread of AstVs between hosts as well as cross-species transmission. For example, if AAstVs do not have increased stability in mucus it would limit their ability to replicate in and spread between mammalian species. Future research exploring the stability of additional MAstVs as well as AAstVs will be critical to determining the factors that promote or mitigate transmission.

As viruses that primarily rely on fecal-oral transmission, the relationship between AstVs and the intestinal environment is an important component to AstV biology. The intestinal environment is a complex array of interactions between the host and the microbes that inhabit it, bacterial and viral alike. Several viruses, both enteric and non-enteric, have been shown to alter the intestinal microbiome during infection [76,77,78]. Initial studies indicate that AstV infection can also alter the microbiome [79]. There is evidence that AstV infection can induce both detrimental and beneficial changes in the intestinal environment. Avian AstVs have been associated with outgrowth of atypical *E. coli*, which causes poultry enteritis mortality syndrome [80,81]. Murine AstV, on the other hand, reduces enteropathogenic *E. coli* colonization by increasing the mucus barrier and mucus-associated bacteria [70]. Murine AstV also protects animals from norovirus and rotavirus infections by modulation of the host antiviral cytokine interferon (IFN) lambda [82]. These studies demonstrate that the impact of AstV infection on the intestines is highly variable between species, with some having an almost commensal effect on the host and others enhancing pathogenic bacteria and death, highlighting the need for additional research on AstV and the microflora in other species.

AstVs are primarily enteric pathogens but have also been found to cause disease outside the gastrointestinal tract. Several AstVs can drive neurological symptoms in both human and non-human mammals, with virus being detectable within the brain and cerebrospinal fluid [3,5,7,40,53]. Studies with HAstVs have also reported detectable virus in the blood and the respiratory tract [3,55,83,84], and another study detected an AstV in nasal swabs from dromedary camels [43]. Whether AstVs are able to transmit from these non-gastrointestinal sites or can only disseminate to these sites from gastrointestinal infection has yet to be determined. A lack of good animal models for AstV infection has hindered the field’s ability to research dissemination and potential transmission outside the gastrointestinal tract. Developing these models will be critical to further understand the biology of neurotropic and other disseminating AstVs.

Much of this review has focused on horizontal transmission, as this is the primary mode in which AstVs have been shown to transmit. However, recent evidence suggests that in avian species, vertical transmission between breeding adults and offspring is also possible. Some strains of chicken AstVs have been detected at high levels in newly hatched chicks [85], in contrast to a previous study that found chicks were negative for AstV at hatching [86]. More recently, a novel AstV in geese was detectable in the vitelline membrane, embryo, and allantoic fluid of unhatched embryos as well as newly hatched goslings [87]. The eggs were collected and incubated after laying, leaving no possibility that the AstV transmitted to the goslings after they were hatched. This, along with the presence of virus inside unhatched and dead embryos, indicates the novel goose AstV (GoAstV) was transmitting vertically. These studies indicate that although the primary mode of transmission of AstV appears to be horizontal, certain strains of AstVs in some species may be able to transmit vertically. No potential vertical transmission has been documented in a mammalian species. The difference between mammalian and avian anatomy could be one reason for this disparity. Aside from monotremes and some marsupials, mammals have separate orifices for the reproductive, urinary, and excretory systems, whereas avian species have cloacae that serve as the only orifice for all three systems. The cloaca represents a unique environment where AAstVs from the excretory system could interact with and enter the reproductive system, permitting vertical transmission. In the case of the novel GoAstV, cloacal swabs from the breeding adults were positive for the virus [87], supporting the idea that this is a site at which vertical transmission could occur. Another key difference between avian and most mammalian species is reproduction through egg laying versus live birth. Only a limited number of viruses are able to cross the placental barrier during pregnancy and infect the fetus [88], adding an additional barrier to vertical transmission. These anatomy differences could explain why there is evidence for vertical transmission in several avian species but none in mammalian species. Vertical transmission of some AAstVs could have serious implications for commercially farmed avian species. The novel GoAstV caused significantly higher rates of mortality in goslings and decreased overall hatchability of eggs from flocks infected with the virus compared to uninfected flocks [87]. Disinfecting and cleaning protocols can reduce horizontal transmission but would have no impact on vertical transmission, making it difficult to prevent or eliminate AstV infection within flocks where vertically transmitting AstVs are present.

## 3. Mutation and Recombination

Like most other RNA viruses, the AstV RdRP is error prone and lacks proofreading capabilities, leading to nucleotide mutations during replication that contribute to genetic diversity [89]. AstVs generate around (3.7 ± 0.1) × 10^−3^ nucleotide substitutions per site per year [90]. Deleterious as well as beneficial mutations can affect viral transmission and evolution. Recombination between different lineages of AstVs can also contribute to viral diversity and evolution. Coinfection of multiple circulating lineages within the same host can facilitate recombination and emergence of novel AstVs within a species [41,59,66]. Although recombination in the ORF1a gene has been shown [57], the ORF1b/ORF2 junction of the AstV genome is the predominant location at which recombination events occur. Consistent with this, multiple recombinant strains of HAstVs have been reported with ORF1b and ORF2 coming from different parental viruses [57,90]. As noted earlier, ORF2 is the most variable region of the genome and encodes the proteins necessary for viruses to enter host cells. Both mutations and/or recombinations at this location have the potential to alter the virus’s ability to attach to and enter host cells. This may be relevant to viral dissemination or cross-species transmission, potentially allowing the virus to use novel cell receptors or adapt to a new species’ receptor and entry machinery. Varying the surface proteins of the virus would also allow it to escape antibody mediated neutralization, as the spike domain is the most common place for neutralizing antibodies to bind [91,92]. The existence of multiple serotypes of AstVs in mammalian and avian species suggests that antibodies drive AstV evolution to evade immunity. Mutations in the HAstV capsid spike allow for escape from neutralizing antibodies [91]. Human clinical studies have also demonstrated that people with AstV antibodies are protected from severe disease and have decreased viral shedding [93,94,95], but the presence of sequential infections in patient populations suggest a lack of heterologous immunity [96]. However, partial neutralization of some spike binding antibodies across serotypes suggests that there are potentially broadly reactive epitopes [91,92]. By acquiring mutations in the spike capsid or promoting recombination of the ORB1b/ORF2 junction, the progeny virus has the potential to increase its chance to enter new cells and avoid existing humoral immunity.

## 4. Cross-Species Transmission

Cross-species transmission to an antigenically naïve population allows for potentially rapid spread and pathogenesis within the new species. Cross-species transmission of both AAstVs and MAstVs have been characterized, with recombination events hypothesized to play a large role (Table 1) [12,18,26,27,57,59,67]. The high density of farm animals that promotes intra-species transmission also provides an environment which favors cross-species transmission of AstV at a high rate and over long periods of time. Both these factors increase the likelihood of a successful cross-species transmission event. Supporting this idea, AstVs detected in farmed guinea fowl had 84.6–100% ORF2 amino acid similarity to turkey astrovirus type 2 (TAstV-2) [35] and AstVs found circulating in domesticated ducks showed ORF1b amino acid similarities to various chicken and turkey AstVs [27]. Proximity of farmed mammalian species has also allowed neurotropic AstVs to cross species barriers. Ovine with encephalitis were infected with an AstV that had an ORF2 with 95–98% nucleotide and amino acid similarity to viruses known to cause encephalitis in bovine [40].

Domestication also has brought humans in close contact with both avian and mammalian AstVs. Reports have shown that some humans who work closely with turkeys have detectable levels of TAstV-2 antibodies [97]. The human, mink, ovine-like (HMO) AstVs that infect humans (Table 1) are more closely related to mink and ovine viruses (40–45% ORF2 amino acid identity) than the classical HAstVs (23–25% ORF2 amino acid identity) [58], suggesting these viruses emerged from these animal reservoirs after close interactions with humans. There is also recent evidence indicating multiple recombination events between porcine AstVs and HAstVs (Table 1) [67]. The directionality of this transmission has been debated, indicating that transmission from humans to livestock could be occurring in addition to traditional zoonosis. It has been proposed that recombination events have also occurred between human and feline AstVs [52]. Together these data demonstrate how the close proximity of humans with other species harboring AstVs can facilitate recombination and cross-species transmission between them.

Whereas other viruses often have to rely on two species being in proximity to each other in order to come into contact with new hosts, the prolonged stability of AstVs in water sources could provide a route to new hosts without them being physically near each other [98]. Studies have identified HAstVs in wastewater, groundwater, and river water [52]. This not only provides means of potential spread among humans via irrigation and domestic water, but also exposure of aquatic species to human viruses. A recently identified novel California sea lion AstV was proposed to have arisen after recombination between an existing California sea lion AstV and a HAstV (Table 1). The novel virus had a HAstV-like polymerase and sea lion AstV-like capsid spike domain, suggesting recombination at the ORF1b/ORF2 junction [18]. Further research sampling various water sources around the globe is required to better characterize both the incidence of AstVs as well as the potential for emergence of new recombinant viruses from those water sources.

As more AstVs are detected and characterized, evidence for recombination events between human and non-human AstVs is increasing. Currently, most studies of AstV recombination and transmission have been identified based on similarity to previously published sequences. More complete understanding of both intra-and inter-species transmission and improved screening of potential sources of novel viruses are required to truly understand the potential for and monitoring of emerging zoonotic AstVs. This is especially critical for neurotropic AstVs, which results in more severe disease.

## 5. Conclusions and Future Research Directions

Advances in deep sequencing have allowed the characterization of AstVs to grow rapidly. This includes the identification of new host species, novel strains in existing species, and the characterization of recombination and cross-species transmission events. Despite these advances, there are still questions fundamental to AstV biology that are critical to further understanding both AstV intra-and inter-species transmission.

The surface receptors that are used for entry into host cells are still not understood. Studies with HAstVs indicate that different serotypes of viruses may use different receptors and co-receptors [99], further complicating our understanding of AstV entry. The cell biology of viral entry is also not fully understood for AstVs. In vitro work with HAstVs has found that virus entry is blunted by drugs affecting clathrin-mediated endocytosis [73]. Whether this is the only mechanism of entry into host cells and if this is utilized by other species of AstVs is still not known. Antibodies against the AstV spike prevent binding to cells, indicating this may be the viral receptor binding domain [91]. Identifying the host surface receptor(s) required for entry and the mechanisms involved, especially in different species of animals, is desperately needed to understand basic AstV biology, how spike mutations affect entry, and the potential for cross-species transmission of different AstVs.

Another barrier to understanding AstV transmission is the lack of knowledge of the cellular tropism of these viruses. However, recent advances using intestinal enteroid cultures has shed some light on human and murine AstV tropisms. All three groups of HAstVs were found to have a multicellular tropism, able to infect mature enterocytes, goblet cells, and intestinal progenitor cells in 3D human intestinal enteroids [68]. Murine AstVs, on the other hand, fail to infect 3D murine intestinal enteroids [69]. To overcome this barrier, the authors took 3D murine enteroid cells and cultured them in 2D transwells under air–liquid interface (ALI) conditions. In this condition, murine AstVs were successfully able to infect and spread between cells apically. Both enterocytes and goblet cells were infected by murine AstV, but intestinal progenitor cells were not [69]. This difference in tropism is likely the reason that the HAstVs could infect the 3D enteroids but the murine AstVs could not. Whether this tropism is conserved across AstVs, especially those infecting avian species, has yet to be determined. Another unanswered question is whether the differences in mammalian and avian body temperature, 37 and 39 °C, respectively, impact viral tropism and the likelihood of a virus to transmit between the two genera. Finally, the tropism and dissemination of neurotropic AstVs has also yet to be well characterized. This knowledge is critical to understanding the most severe disease associated with AstV infections.

Lastly, a major hindrance to AstV research is the lack of good infection models. The development of the intestinal ALI cultures from 3D enteroids has immense potential as a model to explore and better understand various AstVs. Protocols for developing 3D intestinal enteroids already exist for several species infected by AstVs, including pigs, chickens, and bats, and could be applied to the ALI system [100,101,102]. Utilization of the ALI culture from 3D enteroid technique would allow for further studies into AstV cell tropism and mechanisms of cell entry for various species of AstVs. The ALI models would also permit researchers to better study cross-species transmission beyond just comparisons of sequence similarity and phylogeny between viruses. For example, human intestinal ALI cultures could be used to screen viruses from different reservoirs such as bats for zoonotic potential. Additionally, this could allow researchers to explore how different mutations in the genome, especially in ORF2, affect viral transmission and the potential for cross-species spread. The ALI system could also be used to better understand how AstVs activate and interface with the innate immune system. Human and murine AstVs were shown to activate the type III IFN, but not type I IFN [69]. How AstVs are able to evade type I IFN activation is currently unknown. Other non-enveloped, positive-sense RNA viruses such as caliciviruses utilize viral proteins to suppress IFN mRNA production and IRF-3 activation in order to evade the host innate immune response [103]. The ALI model can be utilized to explore what AstVs viral protein(s) to inhibit innate immune activation. Understanding if and how AstVs interface with the innate immune system is critical to understanding viral spread and cross-species transmission, as the innate immune system is the first line of defense to infection.

As novels strains and host species continue to be discovered, understanding AstV transmission and their zoonotic potential is essential. Disseminating HAstVs and AstVs from zoonotic reservoirs remain significant threats to public health. Future research directed at the current gaps in our understanding of AstV infection is critical to understanding how these viruses, especially those that disseminate outside the gastrointestinal tract, transmit within and between different species. Deeper understanding of fundamental AstV biology is needed for tracing and treating infections in humans as well as monitoring emerging viruses with zoonotic potential.

## Figures and Tables

**Table 1:** Avian, mammalian, and unclassified astroviruses by host family and evidence for cross-species recombination.

Host Family	Virus(es)	Reference(s)	Cross-Species Recombination?	Reference(s)
Avastrovirus				
*Accipitridae*	AstV ^1^	[25]		
*Anatidae*	DAstV-1, -2; GoAstV; GNAstV; AstV ^1^	[4,25,26,27,28,29,30]	Yes	[27]
*Ardeidae*	AstV ^1^	[17,25]		
*Columbidae*	PiAstV; ANV	[31,32,33]		
*Monarchidae*	AstV ^1^	[17]		
*Muscicapidae*	AstV ^1^	[17]		
*Numididae*	GFAstV	[34,35]	Yes	[35]
*Phasianidae*	ANV-1, -2; CAstV-1, -2; TAstV-1 to -3	[4,26,36,37,38]	Yes	[12]
*Pycnonotidae*	AstV ^1^	[17,25]		
*Rallidae*	AstV ^1^	[17,25]		
*Scolopacidae*	AstV ^1^	[25,39]		
*Strigidae*	AstV ^1^	[25]		
*Threskiornithidae*	AstV ^1^	[25]		
Mamastrovirus				
*Bovidae*	BoAstV; OAstV	[6,7]	Yes	[6,40,41]
*Camelidae*	DcAstV	[42,43]		
*Canidae*	CaAstV	[44,45]		
*Cervidae*	CcAstV	[46]		
*Chiroptera* ^2^	BtAstV	[47,48,49]		
*Delphinidae*	BdAstV	[18]		
*Felidae*	ChAstV; FeAstV	[19,50,51]	Yes	[52]
*Hominidae*	HAstV-1 to -8; AstV-MLB-1, -2; HMO-AstV-A to -C; AstV-VA-1, -2	[4,53,54,55,56,57,58]	Yes	[52,57,58,59]
*Muridae*	MuAstV-1, -2; RAstV	[60,61,62,63]		
*Mustelidae*	MiAstV	[64]		
*Otariidae*	CslAstV; SslAstV	[18]	Yes	[18]
*Suidae*	PAstV-1 to -5	[65,66,67]	Yes	[67]
Unclassified				
*Actinopterygii* ^3^	AstV ^1^	[21]		
*Diaspididae*	Astro-like virus	[22]		

^1^ AstV detected in animals but a specific virus label has not been assigned. ^2^ AstVs have been found in many species belonging to different families of bats, including *Rhinolophidae*, *Vespertilionidae*, *Emballonuridae*, and *Megadermatidae*. To encompass all families, we noted the *Chiroptera* order.^3^ AstVs have been detected in fish species from several orders of fish, including *Scorpaeniformes* and *Pleuronectiformes*. To denote this, we listed the *Actinopterygii* class. DAstV: duck astrovirus; GoAstV: goose astrovirus; GNAstV: goose nephritic astrovirus; PiAstV: pigeon astrovirus; ANV: avian nephritis virus; GFAstV: guineafowl astrovirus; CAstV: chicken astrovirus; TAstV: turkey astrovirus. BoAstV: bovine astrovirus; OAstV: ovine astrovirus; DcAstV: dromedary camel astrovirus; CaAstV: canine astrovirus; CcAstV: deer astrovirus; BtAstV: bat astrovirus; BdAstV: bottlenose dolphin astrovirus; ChAstV: cheetah astrovirus; FeAstV: feline astrovirus; HAstV: human astrovirus; AstV-MLB: astrovirus MLB; HMO-AstV: human, mink, ovine-like astrovirus; AstV-Va: astrovirus VA; MuAstV: murine astrovirus; RAstV: rat astrovirus; MiAstV: mink astrovirus; CslAstV: California sea lion astrovirus; SslAstV: Stellar sea lion astrovirus; PAstV: porcine astrovirus.
